# Key Amino Acid Substitution for Infection-Enhancing Activity-Free Designer Dengue Vaccines

**DOI:** 10.1016/j.isci.2019.02.012

**Published:** 2019-02-18

**Authors:** Atsushi Yamanaka, Eiji Konishi

**Affiliations:** 1 BIKEN Endowed Department of Dengue Vaccine Development, Faculty of Tropical Medicine, Mahidol University, 420/6 Ratchawithi Road, Ratchathewi, Bangkok 10400, Thailand; 2 BIKEN Endowed Department of Dengue Vaccine Development, Research Institute for Microbial Diseases, Osaka University, 3-1 Yamada-oka, Suita, Osaka 565-0871, Japan

**Keywords:** Physiology, Immunology, Microbiology, Cell Biology

## Abstract

Dengue is a globally important disease caused by four serotypes of dengue virus. Dengue vaccine development has been hampered by antigenic cross-reactivity among serotypes, which potentially causes antibody-dependent enhancement of infection and disease severity. Here we found that a single amino acid substitution in the envelope protein at position 87 from aspartic acid to asparagine or at position 107 from leucine to phenylalanine is critical for suppressing the induction of infection-enhancing antibody in a mouse model. The site and type of amino acid substitution were determined via neutralization escape using an enhancing-activity-only monoclonal antibody that was engineered to reveal neutralizing activity. Mutated dengue type 1 DNA vaccines containing either or both amino acid substitutions induced neutralizing antibodies devoid of enhancing activity against all serotypes. The effect of substitution was further demonstrated using other serotypes and a tetravalent formulation. This finding may contribute to the development of infection-enhancing-activity-free dengue vaccines.

## Highlights

• Amino acids at E87 or E107 are critical for dengue-enhancing antibody induction
• Neutralization escape is useful for identifying the key types or sites of amino acids
• Each substitution can be applied to antigens of all four dengue serotypes
• A modified tetravalent DNA vaccine suppresses enhancing antibody induction in mice

## Graphical Abstract

**Figure:**
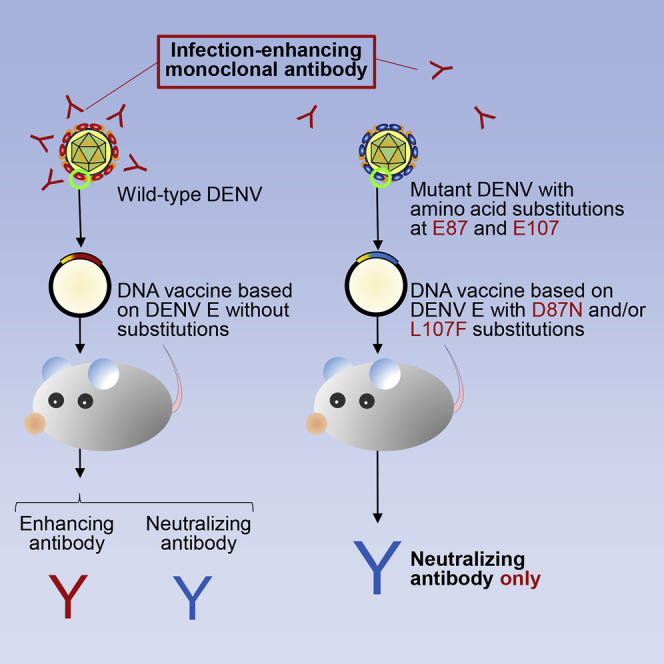

## Introduction

Dengue and severe dengue are globally significant infectious diseases with a wide distribution area (>100 countries) and high patient load (estimated 96 million cases annually) (Guzman and Harris, 2015, World Health Organization, 2017). Attempts at dengue vaccine development have produced attenuated, inactivated, DNA, and subunit vaccines (Scherwitzl et al., 2017). The most advanced dengue vaccine, Dengvaxia by Sanofi, is an attenuated chimeric tetravalent vaccine (Guy et al., 2011). Dengvaxia has been licensed by 20 countries and recommended by the World Health Organization (World Health Organization, 2018); however, its overall protection efficacy is only ∼60% (Sabchareon et al., 2012; Capeding et al., 2014, Villar et al., 2015) and it increased hospitalization rates in some dengue-seronegative populations (Hadinegoro et al., 2015, Halstead, 2016, Dans et al., 2018, Normile, 2017).

Dengue diseases are caused by four antigenically and genetically related serotypes of dengue virus (DENV-1–4: genus *Flavivirus*, family Flaviviridae; Pierson and Diamond, 2013). Flaviviral vaccines have largely been designed to induce neutralizing antibody (NAb), a major protective immunologic factor, against flavivirus infection, thus reducing viremia levels and disease severity (Pierson and Diamond, 2008). Three established flaviviral vaccines for human use (against Japanese encephalitis, yellow fever, or tick-borne encephalitis viruses) can induce NAbs (Ishikawa et al., 2014). However, dengue virus infection, and consequently dengue vaccination, induce infection-enhancing antibody (EAb), as well as NAb. EAb is a serotype-cross-reactive, non-neutralizing antibody; it may be responsible for the disease deterioration that usually occurs upon heterotypic secondary infection (Halstead and O’Rourke, 1977, Halstead, 2003). EAb production has hampered dengue vaccine development and may have reduced the protective efficacy and increased the disease severity in some Dengvaxia vaccines (World Health Organization, 2017, Dans et al., 2018, Normile, 2017). Therefore development of a next-generation dengue vaccine that substantially induces NAb, but not EAb, is needed (Katzelnick and Harris, 2017, Tsai et al., 2017, Rey et al., 2018).

NAb and EAb are induced by three dengue virion surface proteins: premembrane (prM), membrane, and envelope (E). E, the major surface protein, has three domains: EDI, EDII, and EDIII (Modis et al., 2003). EDIII possesses type-specific antibody epitopes, whereas EDII, which contains the fusion loop (FL), possesses cross-reactive ones (Heinz et al., 1982, Roehrig et al., 1998, Roehrig, 2003). Thus use of EDIII alone as a vaccine antigen might work for vaccine improvement (Guzman et al., 2010), but human NAb mainly recognizes quaternary structures like EDI/II (Wahala et al., 2009, de Alwis et al., 2012). Another potential method of eliminating EAb induction is the “knockout” of cross-reactive or EAb epitopes via artificial substitutions of some amino acids contained in vaccine antigens (Crill et al., 2012, Hughes et al., 2012, Tang et al., 2015). However, no vaccine modification design effective enough for all serotypes has been demonstrated yet.

In our previous work, we demonstrated that complement plays an important role in controlling the neutralizing and enhancing activities in human sera and for mouse monoclonal antibodies (Yamanaka et al., 2008, Yamanaka et al., 2012, Yamanaka et al., 2013, Yamanaka and Konishi, 2016, Yamanaka and Konishi, 2017). Specifically, complement-dependent NAbs could maximize their neutralizing activity in the presence of complement, such that the overall outcome is neutralization. In contrast, EAbs have an advantage in the absence of complement. To analyze the final balance between NAbs and EAbs, we included complement in our “antibody assay system for the balance between neutralizing and enhancing activities” (NAb/EAb-balance assay) because doing so should reflect the *in vivo* antibody status more accurately than conducting the assay without complement. Using this system, we previously found that most dengue-immune humans possess complement-independent EAb, the enhancing activity of which did not reduce in the presence of complement (Yamanaka et al., 2012). We also established a mouse monoclonal antibody (mAb) against DENV-1, named D1-V-3H12 (hereafter 3H12), that displayed enhancing but not neutralizing activity, even at a higher IgG concentration (1 mg/mL) (Yamanaka et al., 2013), irrespective of complement inclusion, thus providing a model of complement-independent EAb. Furthermore, we demonstrated using the 3H12 model that NAb-neutralizing activities were suppressed by complement-independent EAb, suggesting a reduction in the protective efficacy of NAb-based vaccines by concomitantly induced EAb (Yamanaka and Konishi, 2016, Yamanaka and Konishi, 2017). Here, we aimed to determine the site and type of amino acid substitutions in a dengue vaccine antigen that are capable of suppressing EAb induction in all serotypes, again using the 3H12 antibody.

## Results

### Search for Critical Amino Acids

**Figure 1:**
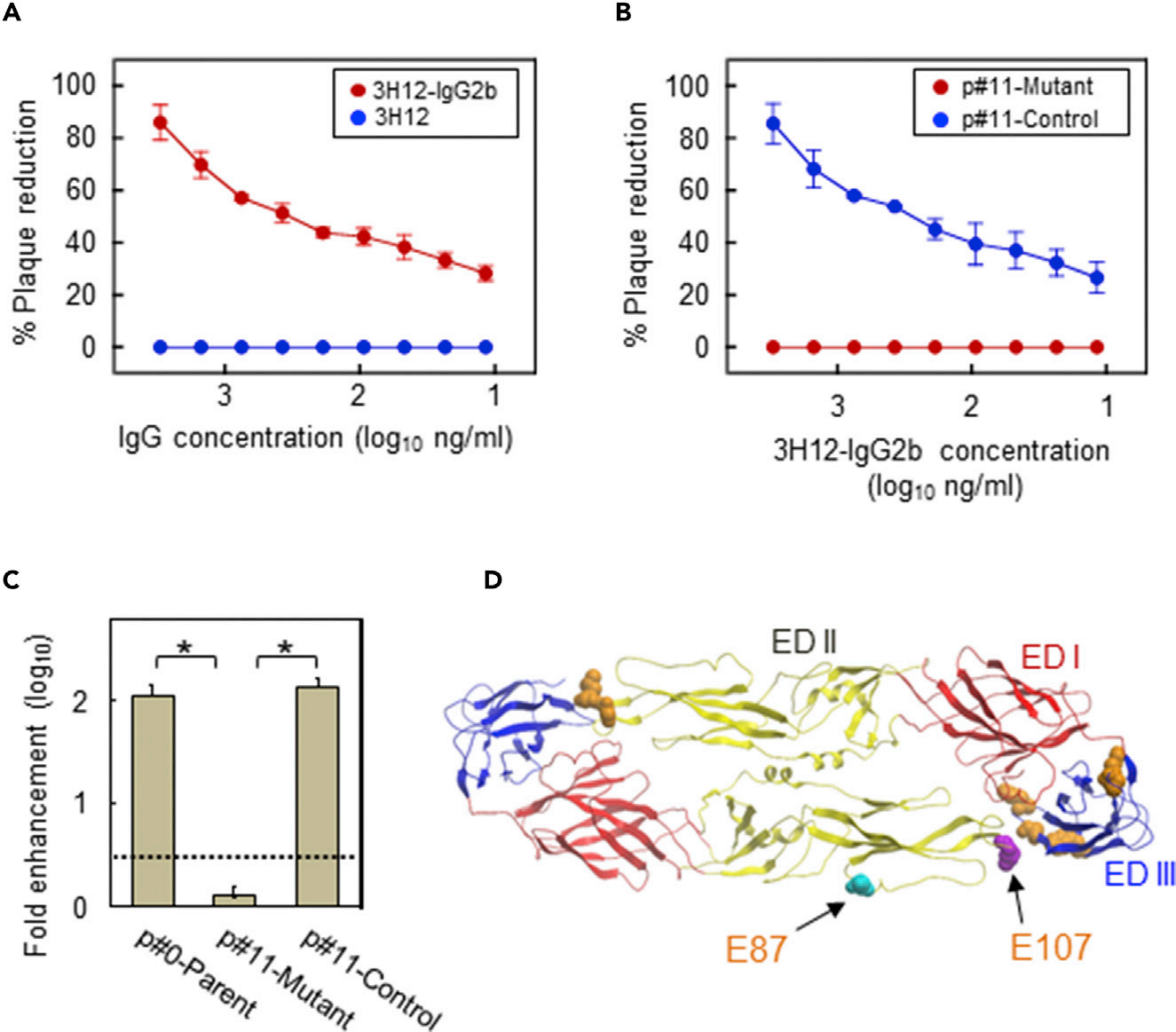
Identification of Amino Acid Substitution Sites in a Neutralization-Escape Mutant (A) Neutralizing activities of 3H12-IgG2b antibody against DENV-1 Mochizuki. The x axis shows the 3H12-IgG2b or 3H12 concentration, and the y axis shows the percentage of plaque reduction. (B) Neutralizing activities of 3H12-IgG2b against an escape mutant obtained after 11 passages (p#11-Mutant) and the corresponding control (p#11-Control). The x axis shows the 3H12-IgG2b concentration, and the y axis displays the percentage of plaque reduction. (C) Infection-enhancing activities of 3H12 against the p#11-Mutant, p#11-Control, and p#0-Parent, expressed as the fold enhancement in the presence of 1 μg/mL of 3H12. Dotted lines indicate the cutoff differentiating enhancing from non-enhancing activities. Data represent averages of two independent assays ± SD (*p < 0.001). (D) Positions 87 and 107 of E (Protein DataBank accession code: 1UZG) with indication of three domains. EDI, EDII, and EDIII are indicated in red, yellow, and blue, respectively. Two key amino acid residues (epitopes) are indicated in green (D87N) and purple (L107F). For reference, a flavivirus-cross-reactive mAb 4G2, which recognizes residues in the E protein, is shown in orange (Crill and Chang, 2004; Chiou et al., 2012) on the other side of the E homodimer.

We applied viral neutralization escape to identify a candidate amino acid substitution that strongly affects the ability of dengue vaccine antigen to induce EAb in a mouse-DNA vaccine model. The mAb 3H12 previously generated from a DENV-1 Mochizuki-immunized mouse and used for obtaining escape mutants has an enhancing-activity-only nature, so we expected that a 3H12-epitope-modified vaccine antigen would not induce 3H12-like antibodies, and this change would thus contribute to a reduction in overall EAb induction. 3H12 targets the E protein, belongs to the IgG1 subclass, and is cross-reactive against all dengue virus serotypes (Yamanaka et al., 2013, Yamanaka and Konishi, 2016). Although 3H12 possesses enhancing activity only, when its subclass was altered to IgG2b (3H12-IgG2b) via molecular engineering, it showed neutralizing activity on Vero cells (Figure 1A).

Eleven passages of the Mochizuki strain through Vero cells in medium containing 3 μg/mL 3H12-IgG2b generated an escape mutant (p#11-Mutant) whose infectivity was neither neutralized by 3H12-IgG2b antibody in the conventional neutralization test using Vero cells (Figure 1B) nor enhanced by the original 3H12 antibody in our NAb/EAb-balance assay using K562 cells (Figure 1C). Nucleotide sequence analyses of the prM/E region revealed that the p#11-Mutant had three differences from the control virus (exposed to 11 passages without 3H12-IgG2b; p#11-Control), whose sequence was identical to that of the parent virus (p#0-Parent). These were (1) from C to A at nucleotide position 4 of prM (deduced amino acid alteration from histidine to asparagine at prM2; H2N), (2) from G to A at nucleotide position 259 of E (aspartic acid to asparagine at E87; D87N), and (3) from C to T at nucleotide position 319 of E (leucine to phenylalanine at E107; L107F). Both E mutations occurred in EDII (Figure 1D). The sites and types of amino acid substitutions found in p#11-Mutant were positively selected under the pressure of neutralizing activity by 3H12-IgG2b; thus, these substitutions were the most suitable candidates for testing their potential to suppress EAb induction by a dengue vaccine.

### Effect in a DENV-1 Mochizuki Model

**Figure 2:**
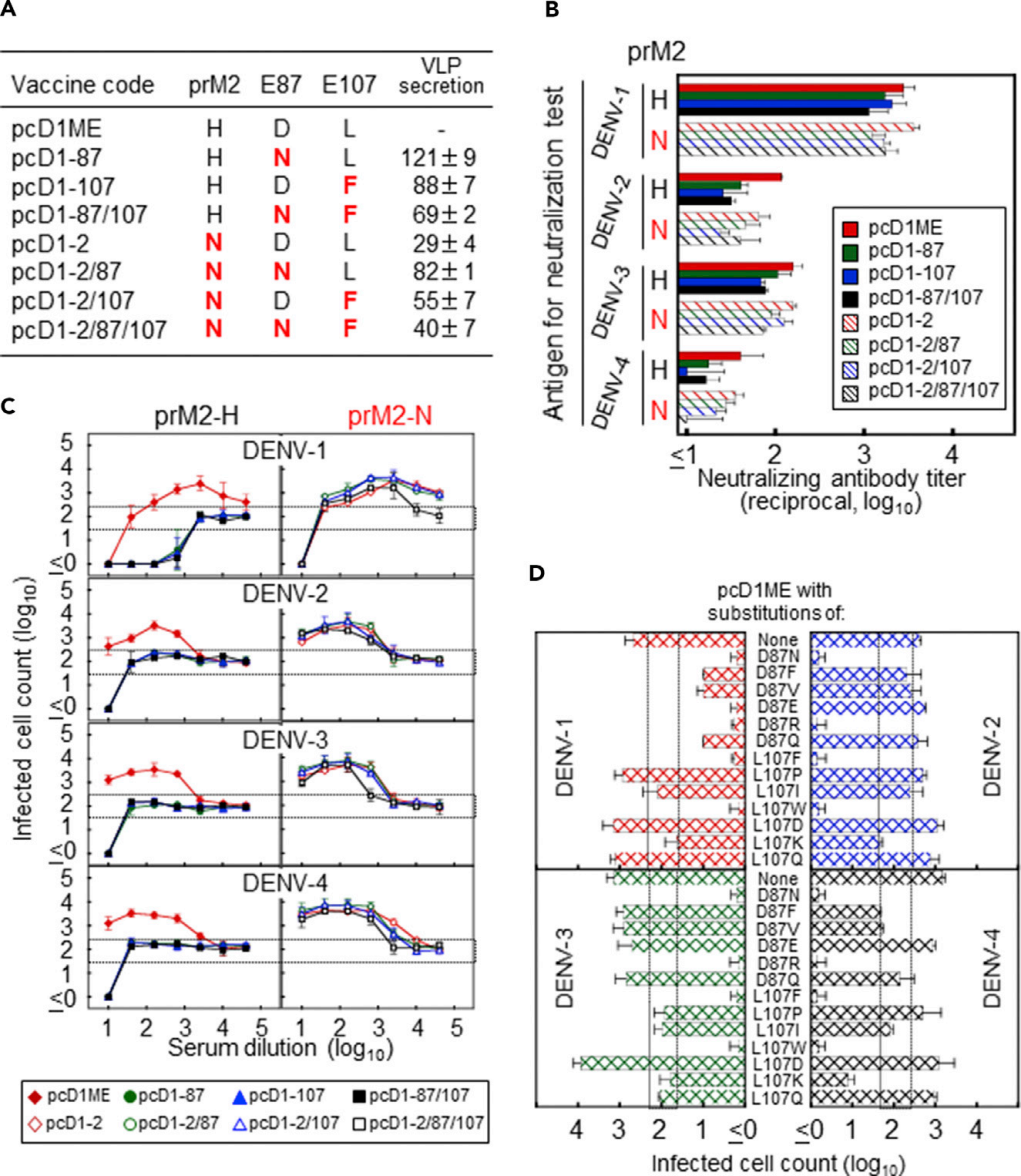
Assessment of EAb Induction Using a DENV-1 Mochizuki-Mouse Model (A) Mutation sites (indicated in red) included in pcD1ME variants. Three amino acids at positions prM2, E87, and E107 were targeted for substitution. The VLP secretion levels of these pcD1ME variants are expressed as relative optical density values (%) compared with that obtained using the original pcD1ME. (B and C) NAb titers (B) and enhancing activities (C) induced in immunized mice. (B) PRNT75 (75% plaque-reduction neutralizing test) antibody titers were obtained from two independent experiments. The x axis shows the serum dilutions corresponding to the PRNT75 value. (C) Enhancing activities were obtained from two independent experiments. The x axis shows the serum dilution, and the y axis displays the infected cell counts (in log_10_). Dotted lines (lower/higher) indicate the cutoff values used to differentiate neutralizing or enhancing activities from non-neutralizing or non-enhancing activities, respectively. (D) Enhancing activities induced by pcD1ME designed to express E containing various single amino acid substitutions at positions 87 or 107. Groups of three mice were used for experiments shown in (D). Enhancing activities are shown as dose-dependent enhancing activity curves in (C) but are shown as values obtained at 1:160 against DENV-1 or 1:10 against DENV-2–DENV-4 in (D). Labels in panels indicate serotypes of assay antigens. Dotted lines indicate the cutoff differentiating neutralizing or enhancing from non-neutralizing or non-enhancing activities. Data represent averages of two independent assays ± SD. See also Figures S1 and S2.

Although the mAb 3H12 is directed against E, we evaluated the effects of all three mutations at prM2, E87, and E107 on antibody responses in a mouse-DNA vaccine model. In this model, the prM/E-gene cassette contained in the plasmid DNA was designed previously to expresses extracellular virus-like particles (VLPs) in inoculated mice (Konishi et al., 2003), providing a strong immunogen that induces reproducible NAb responses (Konishi et al., 2006). To identify which mutation(s) among the three is/are critical for suppressing EAb induction, we used a previously constructed plasmid pcD1ME containing the Mochizuki prM/E genes and constructed variants that included one to three mutations (Figure 2A). The VLP secretion level from 293T cells transfected with these pcD1ME variants containing key amino acid changes was measured by ELISA, and the results were expressed as a ratio relative to the optical density value obtained with the original pcD1ME (Figure 2A). Our data indicate that the VLP secretion level might be negatively regulated by a mutation at prM2, but that it is not obviously suppressed by a mutation at E87 or E107.

BALB/c mice immunized with each plasmid elicited similar NAb titers against DENV-1 (Figure 2B; dose [antibody dilution]-response neutralizing activity curves shown in Figure S1). As expected, the average titers against the homotype (DENV-1) were higher (approximately 10–200 times) than those against heterotypes (DENV-2 to DENV-4).

In contrast, the EAb responses were significantly affected by these mutations (Figure 2C). In our NAb/EAb-balance assay, the number of infected cells following their incubation with virus premixed with antibody specimens was compared with that following cell incubation with virus alone. We regarded antibody activity as enhancing or neutralizing when infected cell counts were significantly higher or lower, respectively, than that obtained with the control. Sera obtained from mice immunized with the unmutated control plasmid (pcD1ME) showed neutralizing and enhancing activities against DENV-1 at low and high dilutions, respectively (Figure 2C, red-filled diamonds in the top left panel). In contrast, sera from mice immunized with pcD1ME containing mutations at E87 or E107 showed no detectable enhancing activity and increased neutralizing activity against DENV-1 (Figure 2C, other symbols in the top left panel). Consistently, enhancing activities against other serotypes were observed in mice immunized with the control plasmid, but not in those immunized with mutated plasmids (Figure 2C, bottom left three panels). Notably, the enhancing activity (1:10 dilution) in control-plasmid-immunized mice turned to neutralizing activity in mutated-plasmid-immunized mice. The dose (antibody dilution)-response antibody activity curves were similar for single and double mutations at E87 and E107. However, when a mutation at prM2 (H2N) was included in the pcD1ME variants (Figure 2C, right panels), enhancing activities were detected, despite the D87N and/or L107F substitution(s) in the E region. These results indicate that the addition of D87N and/or L107F substitution(s) to the Mochizuki E equally abolished EAb induction in mice, but the effect was counteracted by an H2N substitution in prM.

The D87N substitution changed the chemical properties of amino acids in that region from acidic to neutral, whereas the L107F substitution occurred within the non-polar amino acid group. To investigate any relation between amino acid properties at E87 or E107 of the Mochizuki antigen and the ability of the substitution to suppress EAb induction, we selected the following amino acid substitutions to construct mutated plasmids based on pcD1ME: (1) D87F, D87V, L107P, L107I, and L107W for the non-polar amino acid group; (2) D87E and L107D for the acidic amino acid group; (3) D87R and L107K for the basic amino acid group; and (4) D87Q and L107Q for the neutral amino acid group.

Sera from mice immunized with each mutated plasmid were examined in our NAb/EAb-balance assay, and antibody activities at a 1:160 dilution for homologous DENV-1 or a 1:10 dilution for heterologous DENV-2 to DENV-4 are depicted in Figure 2D (the full dataset obtained at 1:10 to 1:40,960 dilutions is shown in Figure S2). Overall, only two amino acid substitutions (D87R, L107W) suppressed EAb induction against all four serotypes at levels equivalent to those obtained with D87N or L107F. For L107F, the same effect was induced by substitution to one (L107W), but not the other two, amino acids (L107P and L107I), within the same amino acid group (non-polar), suggesting the involvement of aromatic rings in the chemical structure of phenylalanine and tryptophan. For D87N, the substitution to arginine, belonging to a different amino acid group (basic; D87R), was similarly effective, but that to glutamine, belonging to the same amino acid group (neutral; D87Q), was not. These results indicate that limited amino acid substitutions can replace D87N and L107F to suppress EAb induction, but effective substitutes do not always relate to the amino acid chemical properties.

### Application to Other Strains or Serotypes

**Figure 3:**
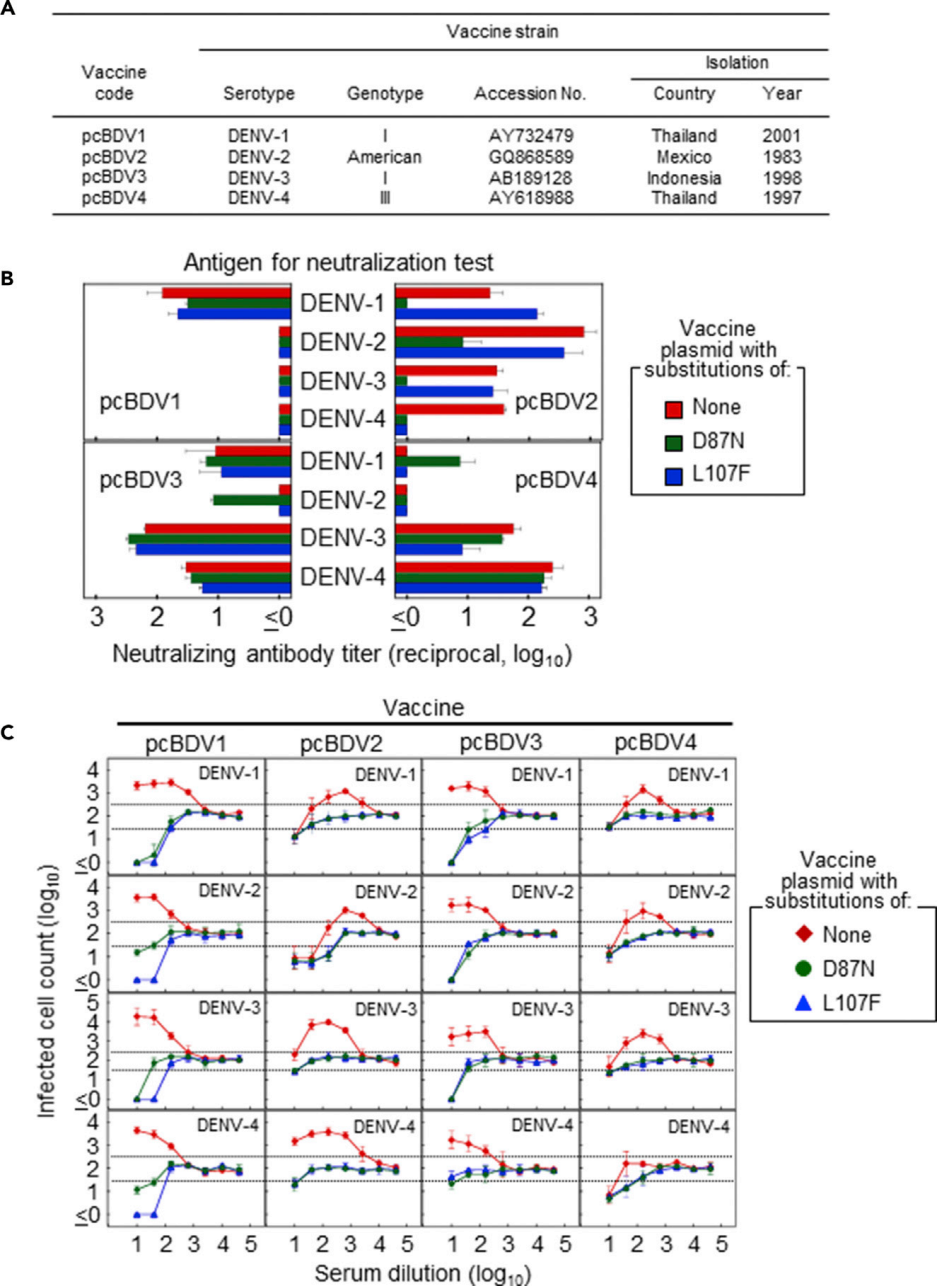
Assessment of EAb Induction Using Strains or Serotypes Other than DENV-1 Mochizuki (A) DENV strains used for constructing DNA vaccines (pcBDV1–pcBDV4) and their variants designed to express E containing amino acid substitutions of D87N or L107F. The genotypes and GenBank accession numbers are provided followed by the countries and years in which the strains were isolated. Each DNA plasmid is defined by the vaccine code of pcBDV-1, pcBDV-2, pcBDV-3, or pcBDV-4; all DNA plasmids were constructed based on the pcDNA3 vector. (B and C) NAb titers (B) and enhancing activities (C) induced in mice (females were used for this experiment) immunized with each of pcBDV1–pcBDV4 or their variants. (B) PRNT75 antibody titers were obtained from two independent experiments. The x axis shows the serum dilutions corresponding to the PRNT75 value. (C) Enhancing activities were obtained from two independent experiments. The x axis shows the serum dilution, and the y axis displays the infected cell counts (in log_10_). Labels in panels indicate serotypes of assay antigens. For dotted lines, see the legend of Figure 2. Data represent averages of two independent assays ± SD. See also Figure S3.

To examine whether the effect of D87N/L107F substitutions demonstrated by using the prototype DENV-1 Mochizuki strain are extended to another DENV-1 (non-prototype) strain and strains of other serotypes, we used the four strains listed in Figure 3A to construct DNA vaccines expressing the E protein with or without the D87N/L107F substitution. Mice immunized with each DNA vaccine induced higher titers of specific NAb against homotypes than titers of cross-reactive NAb against heterotypes (Figures 3B and S3). The addition of a D87N or L107F substitution did not significantly affect the ability of pcBDV1, pcBDV3, or pcBDV4 to induce specific NAb responses. Although the D87N substitution reduced NAb induction in pcBDV2-immunized mice, it did not affect evaluation of EAb responses because the enhancing activity can be detected with higher sensitivity in the NAb/EAb-balance assay (Figure 3C). Consistent with the result obtained using the Mochizuki strain, any DNA vaccine with the D87N or L107F substitution failed to display enhancing activity against any serotype. These results indicate that the D87N/L107F substitution can broadly suppress the EAb responses induced by DNA vaccines against all DENV serotypes in mice.

### Effect on a Tetravalent Vaccine

**Figure 4:**
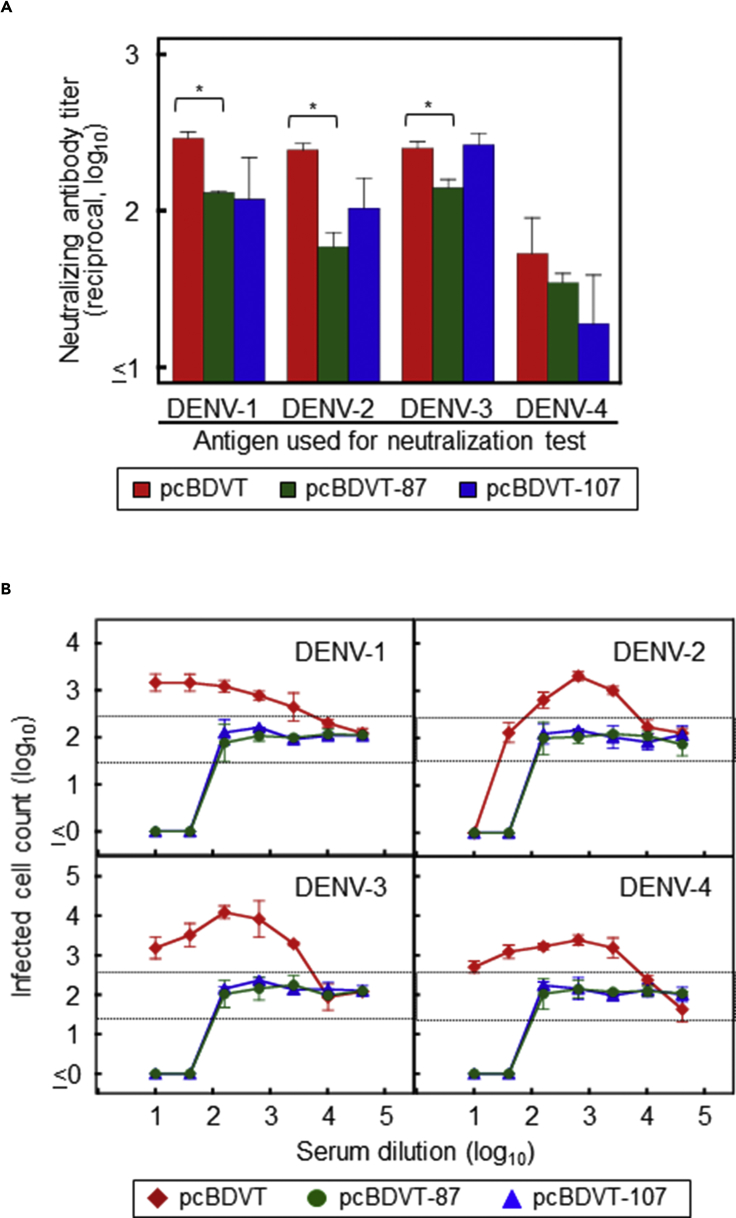
Assessment of EAb Induction Using a Tetravalent Vaccine (A and B) NAb titers (A) and enhancing activities (B) induced in mice immunized with a tetravalent formulation of pcBDV1–4 (pcBDVT) or their variants designed to express E containing amino acid substitutions of D87N (pcBDVT-87) or L107F (pcBDVT-107). (A) PRNT75 antibody titers were obtained from two independent experiments. The x axis shows the serum dilutions corresponding to the PRNT75 value. Asterisks indicate significant differences (p< 0.05) from the PRNT75 values obtained for pcBDVT. (B) Enhancing activities were obtained from two independent experiments. The x axis shows the serum dilution, and the y axis displays the infected cell counts (in log_10_). Labels in panels indicate serotypes of assay antigens. For dotted lines, see the legend of Figure 2. Data represent averages of two independent assays ± SD. See also Figure S4.

To examine if the tetravalent formulation of the DNA vaccine may influence the suppressive effect of D87N or L107F substitution on EAb induction, four vaccines against different serotypes were evenly mixed to prepare a tetravalent DNA vaccine; vaccines without or with D87N or L107F were designated pcBDVT, pcBDVT-87, or pcBDVT-107, respectively. Although a tetravalent DNA vaccine of pcBDVT-87 induced significantly lower NAb titers against DENV-1, DENV-2, and DENV-3 compared with the original pcBDVT (p < 0.05), there were <4-fold differences among the NAb titers induced by pcBDVT, pcBDVT-87, and pcBDVT-107 against each serotype (Figures 4A and S4). Consistent with the results shown in Figures 2C and 3C, enhancing activities induced by pcBDVT were not observed in mice immunized with pcBDVT-87 or pcBDVT-107 (Figure 4B). These results indicate that the suppressive effect of the D87N/L107F substitution on EAb induction was maintained even in the tetravalent formulation.

### 3H12-Binding Site

**Figure 5:**
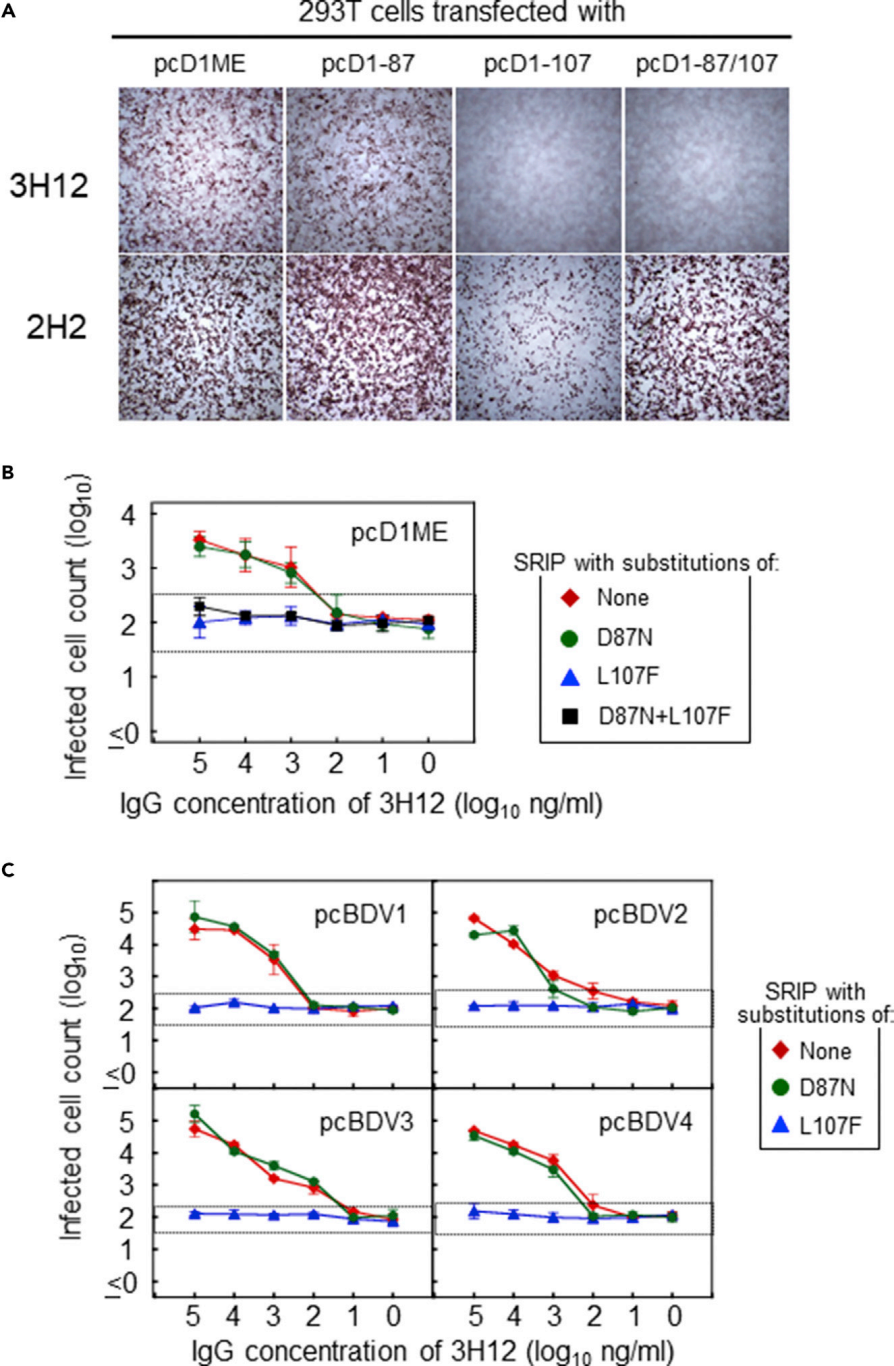
Determination of 3H12-Binding Site (A) Micrographs of 293T cells transfected with pcD1ME or its variants. At 24 h after transfection, the cells were fixed and immunostained with 3H12 or 2H2. (B and C) Enhancing activities of 3H12 against SRIPs generated using pcD1ME variants (B) or pcBDV1–4 variants (C) designed to contain amino acid substitutions of D87N and/or L107F. The x axis shows the 3H12 IgG concentration, and the y axis displays the infected cell counts (in log_10_). For dotted lines, see the legend of Figure 2. Data represent averages of two independent assays ± SD.

To identify the 3H12-binding site, we compared the reactivities of 3H12 to the Mochizuki E antigen expressed with and without the D87N/L107F substitution. 3H12 bound to cells transfected with pcD1ME or pcD1-87, but not to those transfected with pcD1-107 or pcD1-87/107. In contrast, a reference antibody, 2H2 (prM-specific, flavivirus group cross-reactive), bound to cells transfected with any of these plasmids, although the binding of 2H2 to cells transfected with pcD1-107 was comparatively weak (Figure 5A). Next, we used single-round infectious particles (SRIPs) designed to contain E with or without a D87N/L107F substitution on their surface, which were generated by co-transfecting cells with replicon plasmid pCMV-JErep-fullC (Yamanaka et al., 2014) and prM/E-expressing plasmids (pcD1ME, pcD1-87, pcD1-107, or pcD1-87/107). The NAb/EAb-balance assay revealed that 3H12 displays enhancing activity against SRIPs generated using pcD1ME or pcD1-87, but not against those generated using pcD1-107 or pcD1-87/107 (Figure 5B). These results indicate that 3H12 can directly bind an antigen epitope containing the E107, but not E87, of the DENV-1 Mochizuki E.

As 3H12 is a cross-reactive antibody, we next examined if 3H12 could recognize the E107 of other serotypes by using SRIPs generated using pcBDV1 to pcBDV4 and their variants containing the D87N or L107F substitution. The enhancing activity of 3H12 was abolished by the L107F, but not the D87N, substitution (Figure 5C). These results indicate that 3H12 consistently recognizes the epitope containing E107, but not E87, in all four serotypes.

### Effect on IgG Subclass Antibody Response

**Figure 6:**
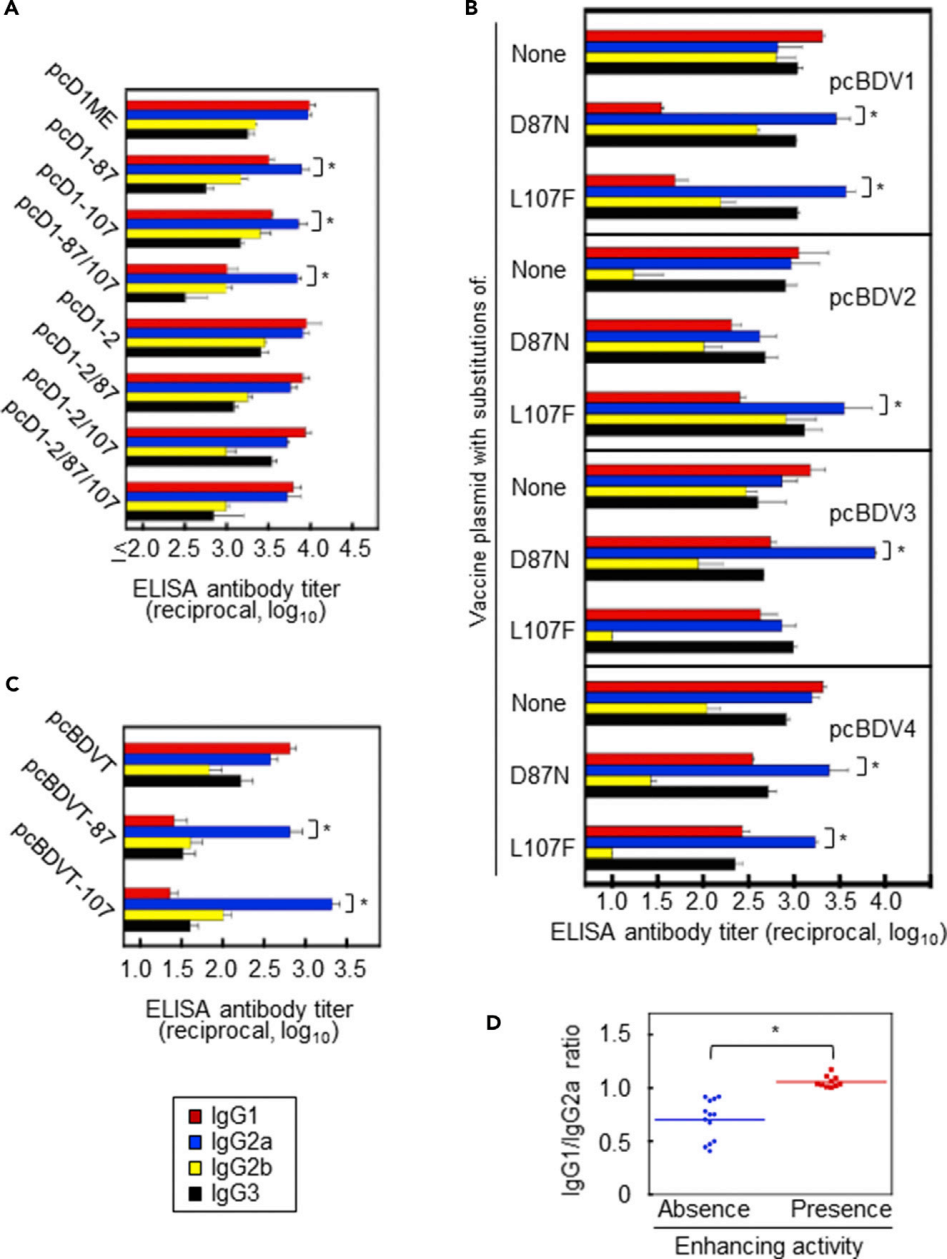
IgG Subclass Antibody Levels in Mice Immunized with Mutated DNA Vaccines (A–C) ELISA antibody titers of each IgG subclass against the DENV-1 Mochizuki strain were determined using the mouse serum samples obtained in (A) Figure 2, (B) Figure 3, and (C) Figure 4. The endpoint titer is expressed as the maximum serum dilution that displayed an optical density (OD) value of ≥0.5. For cases in which the OD value was <0.5 at the 1:20 serum dilution, the titer was defined as 1:10. Mouse IgG1, IgG2a, IgG2b, and IgG3 are shown in red, blue, yellow, and black, respectively. Asterisks indicate significant differences (p< 0.05) between IgG1 and IgG2a levels. (D) Summary of the IgG1/IgG2a ratios in mouse sera, divided into groups based on the presence or absence of enhancing activity. The asterisk indicates a significant difference (p< 0.05).

To investigate the mechanism involved in the suppression of EAb induction by the D87N substitution, we compared antibody levels among IgG subclasses contained in the mouse sera obtained in the experiments shown in Figures 2, 3, and 4, using an ELISA with the DENV-1 Mochizuki antigen. As shown in Figure 6A, sera that failed to show enhancing activity (mice immunized with pcD1-87) had higher levels of IgG2a antibody than of IgG1 antibody (p< 0.05), whereas sera that showed enhancing activity (mice immunized with pcD1ME, pcD1-2, or pcD1-2/87) did not (p> 0.05). Similar results were observed for mice immunized with monovalent or tetravalent formulations of pcBDV1–pcBDV4 (Figures 6B and 6C), except for those immunized with the monovalent formulation of pcBDV2-D87N (p = 0.17) or with the monovalent formulation of pcBDV3-L107F (p = 0.30). In addition, the L107F substitution also decreased the ratio of IgG1/IgG2a antibody levels. Ultimately, the IgG1/IgG2a ratios were significantly lower in mouse sera that displayed an absence of enhancing activity (p< 0.05) compared with those in mouse sera that showed the presence of enhancing activity (Figure 6D). These results indicate that mice immunized with a DNA plasmid including D87N/L107F substitutions showed lower ratios of IgG1:IgG2a antibody responses.

## Discussion

Our attempt to allow dengue virus to escape from an enhancing-activity-only antibody succeeded in identifying the sites and types of key amino acids responsible for EAb induction in mice. One group previously identified multiple amino acid modifications of the E protein (G106R, L107D, K310D/E, E311K/R, and P364Q/R) based on bioinformatics and demonstrated suppressed induction of serotype cross-reactive antibody in mice using DENV-1 and DENV-2 (Crill et al., 2012, Hughes et al., 2012), whereas another group modified an EAb epitope at E8 (N8R) reactive to two enhancing-activity-only mAbs and evaluated its effect in a mouse DENV-2 challenge model (Tang et al., 2015). Here we demonstrated that a single amino acid substitution in E (D87N or L107F) suppressed EAb induction for all four DENV serotypes. A major advantage of the vaccine design approach used in the present work is the simple achievability of a single mutation. Furthermore, a minimized number of modifications (e.g., a one-point modification) in a vaccine antigen might allow the antigen to maintain its immunogenicity in a mouse model, even if the key amino acid modification was applied to other DENV strains and other vaccine platforms.

E107 is located in the FL region of EDII. This region is highly conserved among flaviviruses, and the amino acid at E107 is almost exclusively leucine (Allison et al., 2001). Interestingly, the only exceptions, Powassan virus and deer tick virus of the tick-borne encephalitis virus group, have phenylalanine at E107 (Crill and Chang, 2004), indicating that the amino acid found in E107 in our study is, although limited, used in nature. The FL region has been a target of antigen modification for suppressing induction of DENV serotype-cross-reactive antibodies, even in a recently developed Zika vaccine candidate, although the L107F substitution has never been reported (Crill et al., 2012, Hughes et al., 2012, Richner et al., 2017). Among DENV serotype-cross-reactive antibodies, including those against prM, antibodies against the FL region are one of the major components in dengue-immune mouse and human sera (Crill et al., 2012, Hughes et al., 2012, de Alwis et al., 2014, Lai et al., 2008). This provides one possible explanation for the successful reduction in EAb induction down to undetectable levels by a single amino acid substitution in the FL region of the DENV E antigen.

In contrast, E87 is located on the lateral ridge of EDII. Although we failed to demonstrate the direct binding of 3H12 to E87, the D87N substitution that occurred during the process of escaping from the neutralizing activity of 3H12-IgG2b still suppressed EAb induction in mice. The detailed mechanism remains to be identified, but higher levels of IgG2a antibody than of IgG1 antibody were observed in mice immunized with D87N-substituted DNA variants. We compared IgG1 and IgG2a antibody levels because each IgG subclass has different complement-binding affinity (IgG2a ≈ IgG2b > IgG3 > IgG1 in mice) (Baudino et al., 2006), and, thus, the total enhancing activity in sera detected by our NAb/EAb-balance assay including complement depends on the antibody levels of each subclass, especially IgG1 and IgG2a. As lower and higher ratios of IgG1:IgG2a antibody levels relate to Th1- and Th2-type immune responses in mice, respectively (Finkelman et al., 1990, Stavnezer and Amemiya, 2004), the D87N substitution may have contributed to the induction of Th1-type immune responses. Amino acid sequences have been reported to affect Th-type immune responses (Evavold and Allen, 1991), and we previously observed various IgG1:IgG2a antibody responses induced by different DENV strains in mice (Sjatha et al., 2013). However, an indirect relation of E87 to E107 in the E structure is not ruled out, because E87 is also well conserved in DENV.

An amino acid substitution at prM2 (H2N) was also found in the Mochizuki variant escaping from the E-directed antibody 3H12-IgG2b. Although prM contributes to the proper intracellular maturation of E (Roby et al., 2015), the specific function of the amino acid at prM2 is unknown. Interestingly, the H2N substitution counteracted the effects of the D87N and/or L107F substitution(s) in the Mochizuki E antigen on the suppression of EAb induction in mice. This demonstrates that a certain amino acid in prM weakens the effects of D87N/L107F in any other DENV strain. However, the substitution at E87 or E107 worked for suppressing EAb induction without altering the original prM sequence in four DENV strains, in addition to Mochizuki, in our mouse-DNA vaccine model. Further studies are needed to elucidate the mechanisms underlying the suppressed EAb induction caused by D87N/L107F substitutions and the counteracted effect of D87N/L107F caused by H-prM2-N substitution.

In conclusion, we demonstrated that the E antigen of any DENV serotype with the D87N/L107F substitution can induce enhancing-activity-free NAb in mice. Suppression of EAb induction may maximize vaccine efficacy and minimize the potential risk of vaccine-induced enhancement of DENV infection. In our future work, we plan to expand our investigations of key amino acid modifications into vaccine candidates for other flaviviruses, such as Zika and West Nile viruses.

### Limitations of the Study

As with any work, the present study has limitations. First, all work was conducted using only a single mouse model, and the enhancing activity was tested only *in vitro*. Thus for the development of a safe and effective designer dengue vaccine candidate containing the D87N and/or L107F substitution(s) in the E antigen, further studies using a DENV challenge animal model, such as AG129 mice, will be required to investigate the *in vivo* protection efficacy. Second, because K562 cells have Fc gamma receptor II but no other receptors associated with generating enhancing activity, the dengue infection enhancement phenomenon shown in the present study was all Fc gamma receptor II mediated. To determine if other Fc gamma receptors also mediate enhancing activity to dengue vaccine candidates with D87N or L107F substitution(s) in the E antigen, other assays using various cell lines (such as U937, THP-1, and HL60 cells) will be required. Third, we confirmed the durability of suppressed EAb induction at 8 weeks post-vaccination only; further work is needed to determine if designer vaccines with these modifications could confer lifetime protection. It is important to monitor the safety signal over a long period, given that the recent problem with Dengvaxia initially occurred several years after the first vaccination. Therefore for the development of our findings into a safe dengue vaccine, further animal model studies over a long period will be needed to observe the antibody condition and any associated changes over time.

## Methods

All methods can be found in the accompanying Transparent Methods supplemental file.

## Supplemental Information

Document S1. Transparent Methods and Figures S1–S4
