# Supplementary material for: Key Amino Acid Substitution for Infection-Enhancing Activity-Free Designer Dengue Vaccines
Source: iScience. 2019 Feb 18;13:125–37. doi: 10.1016/j.isci.2019.02.012 (PMC6402262; doi:10.1016/j.isci.2019.02.012)
Supplement: Document S1. Transparent Methods and Figures S1–S4 [file mmc1.pdf]

ISCI, Volume *13*

Supplemental Information

Key Amino Acid Substitution for Infection-Enhancing Activity-Free Designer Dengue Vaccines

Atsushi Yamanaka and Eiji Konishi

Figure S1

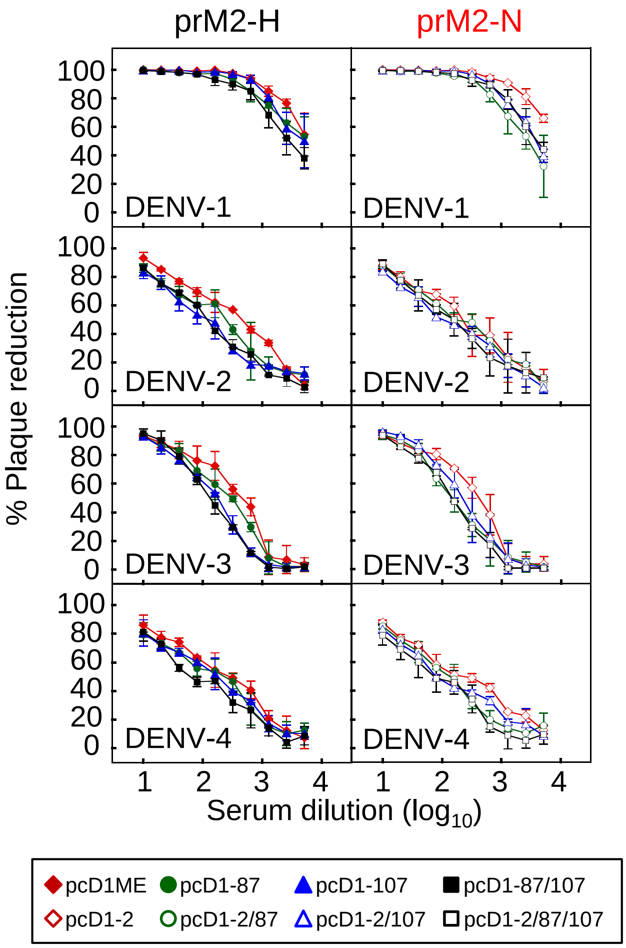

Figure S1. Dose-dependent neutralizing activity curves obtained with sera from mice immunized with pcD1ME or its variants, related to Figure 2. The vaccine codes correspond to those in Fig. 2A. Labels in panels indicate serotypes of assay antigens. Data represent averages of two independent assays ± SD.

Figure S2

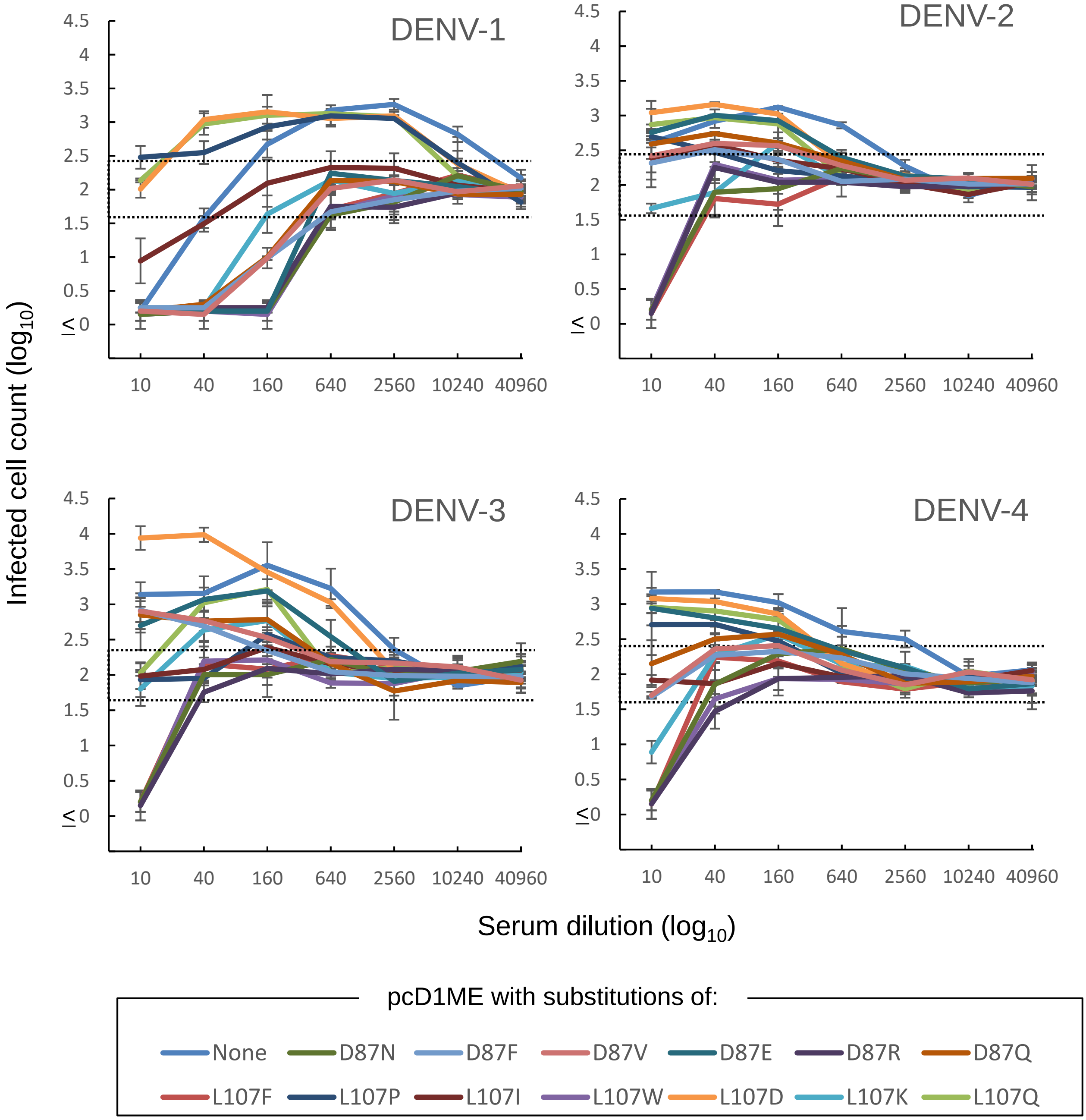

Figure S2. Dose-dependent enhancing activity curves obtained with sera from mice immunized with pcD1ME or its variants, related to Figure 2. Labels in panels indicate serotypes of assay antigens. Data represent averages of two independent assays ± SD. Dotted lines indicate the cut-off differentiating neutralizing/enhancing from non-neutralizing/non-enhancing activities.

Figure S3

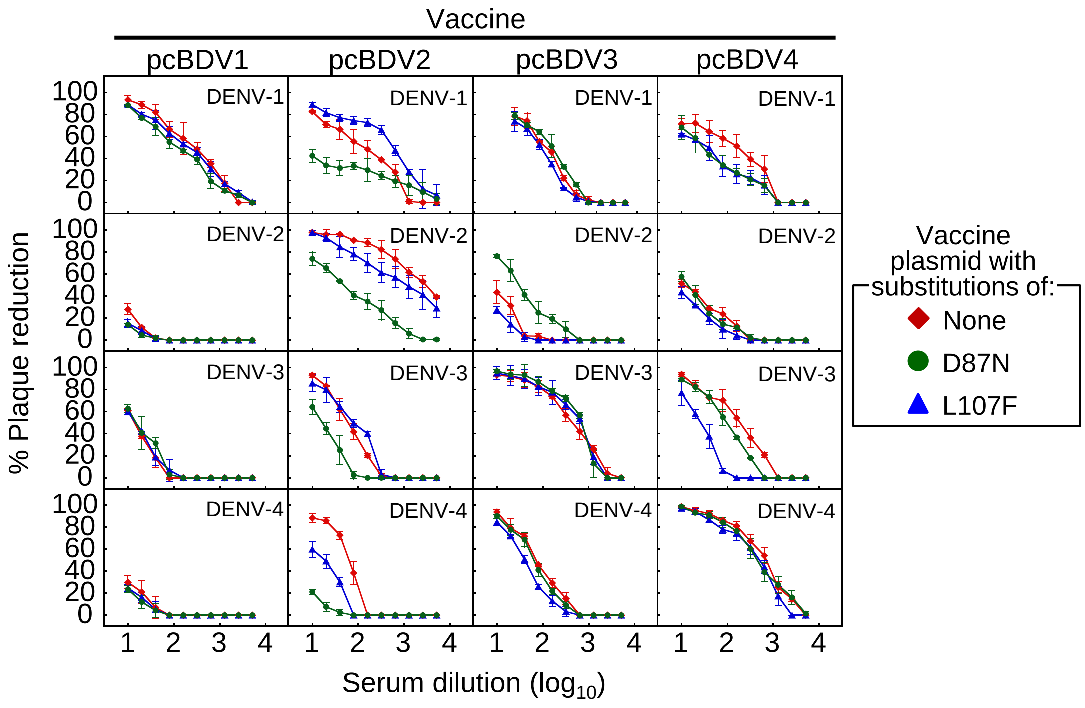

Figure S3. Dose-dependent neutralizing activity curves obtained with sera from mice immunized with pcBDV1–pcBDV4 or their variants, related to Figure 3.

Labels in panels indicate serotypes of assay antigens. Data represent averages of two independent assays ± SD.

Figure S4

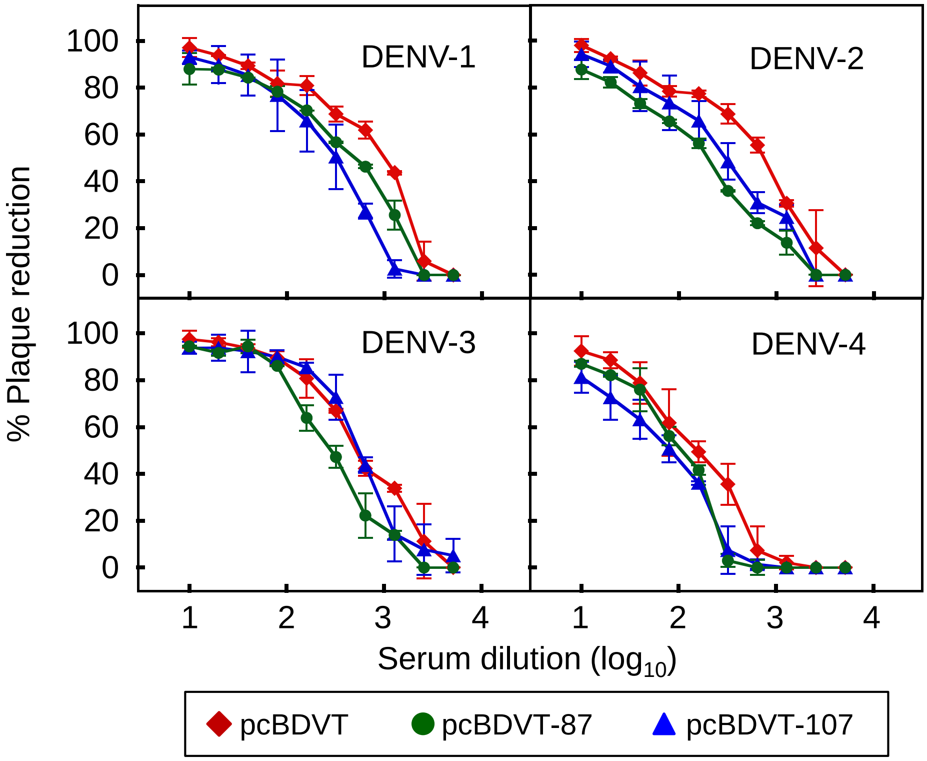

Figure S4. Dose-dependent neutralizing activity curves obtained with sera from mice immunized with a tetravalent vaccine, related to Figure 4. The tetravalent vaccine was composed of pcBDV1–4 (pcBDVT) or their variants designed to express E containing amino acid substitutions of D87N (pcBDVT-87) or L107F (pcBDVT-107). Labels in panels indicate serotypes of assay antigens. Data represent averages of two independent assays ± SD.

Transparent Methods

Ethics statement.

This study was conducted according to the Guidelines for Animal Experimentation at the Faculty of Tropical Medicine of Mahidol University and the Research Institute for Microbial Diseases of Osaka University. The protocol was approved by the Animal Care and Use Committees at the Faculty of Tropical Medicine of Mahidol University (Protocol No. 003-2015) and the Research Institute for Microbial Diseases of Osaka University (H27-09-0).

Mouse experiments

Groups of six 6-week-old male BALB/c mice (25–30 g bodyweight; National Laboratory Animal Center, Mahidol University, Bangkok, Thailand) were used unless otherwise stated. Mice were given three doses (100 µg/dose) of plasmid DNA at 3-week intervals by inoculation into their anterior tibial muscles with an electroporator (NEPA21; Nepa Gene) set to the following conditions: a poring pulse (voltage, 100 V; pulse interval, 50 ms; pulse length, 30 ms; number of pulses, 3; 10% decay rate with + polarity) and a transfer pulse (voltage, 20 V; pulse interval, 50 ms; pulse length, 50 ms; number of pulses, 5; 40% decay rate with ± polarity). The tetravalent formulation was prepared by mixing 25 µg of each of four plasmids of different serotypes (totalling 100 µg). Retroorbital blood was collected from each mouse two weeks after the third immunization, and pooled sera were examined for the neutralization tests, NAb/EAb-balance assays, and ELISAs for measuring the IgG subclass antibody levels.

Cells

African green monkey kidney Vero cells were cultivated in Eagle's minimum essential medium supplemented with 10% fetal bovine serum (FBS) and 60 µg/mL kanamycin (Konishi and Fujii, 2002). Human erythroleukemia K562 cells were cultivated in RPMI 1640 medium supplemented with 10% FBS, 100 units/mL penicillin, and 100 µg/mL streptomycin (Yamanaka et al., 2008). Human embryonic kidney 293T cells were cultivated in Dulbecco's modified Eagle's medium supplemented with 10% FBS (Yamanaka et al., 2014). All cell lines were cultivated in a humidified atmosphere of 5% CO_2:95% air at 37 °C.

Viruses

The DENV-1 (Mochizuki strain), DENV-2 (New Guinea C [NGC] strain), DENV-3 (H87 strain), and DENV-4 (H241 strain) used in this study have been described previously (Konishi et al., 2006). The culture fluids harvested from infected Vero cells were used as live virus sources for the neutralization test, antibody assay for the balance between neutralizing and enhancing activities (NAb/EAb-balance assay), and the generation of a neutralization escape mutant viruses; they were also used as the antigen for enzyme-linked immunosorbent assays (ELISAs).

Monoclonal antibodies (MAbs)

D1-V-3H12 (3H12) was previously generated from a mouse immunized with the DENV-1 Mochizuki strain (Yamanaka et al., 2013). The 3H12-IgG2b antibody was produced as

previously described (Yamanaka et al., 2013). Briefly, 293T cells were co-transfected with two antibody-expressing plasmids (pFUSE series; InvivoGen) encoding the variable region of D1-V-3H12 and the constant region of the heavy (IgG2b) or light (κ) chain and incubated at 37 °C for 3–6 days before culture fluids containing 3H12-IgG2b were harvested. JE-2D5, a mouse MAb specific for Japanese encephalitis virus (JEV) non-structural protein 1, was used to immunostain cells infected with single-round infectious particles (SRIPs) (Konishi et al., 2004) (see below). The 4G2 (E-specific, flavivirus-group-cross-reactive) and 2H2 (prM-specific, flavivirus-group-cross-reactive) antibodies were purchased from the American Type Culture Collection, and JE-10B4 (flavivirus-group-cross-reactive: hereafter 10B4) (Konishi et al., 2008) was used to immunostain cells infected with viruses or transfected with prM- and E-expressing plasmids (see below).

Plasmids

A pcDNA3-based plasmid expressing the prM and E of the DENV-1 prototype Mochizuki strain (pcD1ME) was constructed previously (Konishi et al., 2006). With pcD1ME as a template, site-directed mutagenesis was performed on the amino acid residues at position 2 of the prM protein to replace histidine with asparagine (H2N), at position 87 of the E protein to replace aspartic acid with asparagine (D87N), and at position 107 of E to replace leucine with phenylalanine (L107F), using the KOD Mutagenesis kit (TOYOBO, Japan) according to the manufacturer’s instructions. Primer sets used for the substitutions H2N, D87N, or L107F were, respectively: H2Nfw (5′-AATCTGACCACACGAGGGGAGA-3′) and H2Nrv (5′-GAACGCCAGGGCTGTGGGCA-3′); D87Nfw (5′-

CAAAACGCGAACTTCGTGTGTCGACG-3′) and D87Nrv (5′-TTCTTCCACCAGTGTGGCTTCTCCT-3′); or L107Ffw (5′-GGGTTTTTCGGAAAAGGTAGCCTAAT-3′) and L107Frv (5′-ACAGCCATTGCCCCAGCCTC-3′), where the underlined nucleotides indicate those designed to introduce amino acid substitutions. Proper substitutions were confirmed by sequencing the whole prM/E region of every construct. pcD1ME variants containing other amino acid substitutions at E87 or E107 that were used for the experiments shown in Figure 2D were constructed using pcD1ME as a template and a site-directed mutagenesis service provided by GenScript. Other pcDNA3-based plasmids expressing the prM and E of the non-prototype DENV-1 to DENV-4 strains (pcBDV1 to pcBDV4 used for the experiments shown in Figure 3) were constructed using DNA fragments corresponding to the prM/E region synthesized by GenScript based on nucleotide sequence information available in GenBank. Nucleotide and amino acid sequences in the prM/E regions of all plasmids used in this study are available from the authors upon request.

Generation and analysis of the neutralization escape mutant virus

Vero cell monolayers prepared in a six-well plate were inoculated with the DENV-1 Mochizuki strain (defined as “p#0-Parent” in this experiment) at a multiplicity of infection of 0.1 and incubated at 37 °C in medium containing the 3H12-IgG2b antibody at a concentration of 3 µg/mL. The culture fluid harvested from the first round of incubation was used for a second-round infection of cells, and a similar passage was performed weekly. A control Vero cell culture was infected in the same manner but was maintained without antibody. Viral RNA was extracted

from the culture fluid, and the 498 and 1,485 nucleotides of the prM and E regions, respectively, were sequenced. In the present study, we used viruses contained in the culture fluids after eleven passages in the presence or absence of the 3H12-IgG2b antibody (defined as “p#11-Mutant” or “p#11-Control”, respectively). The substitution sites contained in the p#11-Mutant were identified by comparing its nucleotide and deduced amino acid sequences with those contained in the p#0-Parent and p#11-Control.

Neutralization test

The Vero cell plaque reduction neutralization tests of the antibody specimens (MAbs or pooled sera) were performed with DENV-1 to DENV-4, essentially as described previously (Konishi et al., 2003). Briefly, mixtures of (i) the virus, (ii) two-fold serial dilutions of antibody samples (starting from 1:10 for sera), and (iii) rabbit complement at a final concentration of 5% were incubated at 4 °C overnight. Vero cell monolayers prepared in a 24-well microplate were inoculated with the virus–antibody mixture and incubated at 37 °C for 3 days. After fixation and immunostaining using 4G2 or 10B4 antibody, the plaques were counted. The neutralizing activities are expressed as percentages of plaque reduction calculated relative to the results for virus controls without test samples. The NAb titers are expressed as the maximum serum dilution showing ≥75% plaque reduction, which was determined by the FORECAST function in Microsoft Excel (Microsoft Corporation). For cases in which the NAb titer was lower than 1:10, the titer was defined as 1:5.

Antibody assay for the balance between neutralizing and enhancing activities (NAb/EAb-balance assay)

The balance between neutralizing and enhancing activities of the antibody specimens (3H12 or pooled sera) was measured with semi-adherent K562 cells and expressed as the number of infected cells, as described previously (Konishi et al., 2010). Briefly, serial dilutions of antibody specimens were mixed with DENV-1–4 or SRIP antigens (see below) and incubated at 37 °C for 2 h in the presence of rabbit complement at a final concentration of 5%. K562 cells were then added to the mixtures and incubated at 37 °C for 2 days. After fixation and immunostaining using 4G2 or 10B4 antibody, the infected cells were counted. The cut-off values for neutralizing and enhancing activities were calculated from the means ± three standard deviations (SD) of infected cell counts obtained with eight negative controls adjusted for approximately 100 infected cells. When the number of infected cells was higher than the mean + three SD, it was defined as enhancing activity. In contrast, when the infected cell numbers were lower than the mean − three SD, it was defined as neutralizing activity. The fold enhancement was calculated from the number of infected cells obtained at one dilution of antibody (10 μg/mL of 3H12) and was expressed in $\log_{10}$ as the increase in the infected cell count relative to the negative control.

Immunostaining

Immunochemical staining was performed essentially as described previously (Konishi and Fujii, 2002). For staining infected cells in antibody assays, cells were fixed and incubated

serially with a MAb (4G2 for DENV-1–4 or JE-2D5 for SRIP antigens), biotinylated anti-mouse IgG, ABC (avidin-biotinylated peroxidase complex) reagent, and VIP substrate (Vector Laboratories). For experiments to identify the binding site of 3H12, 293T cell monolayers prepared in wells of 24-well plates were transfected with 500 ng of pcD1ME or its variants, using Lipofectamine LTX and Plus Reagent (Invitrogen), according to the manufacturer's instructions. After being incubated at 37 °C for 24 h, the cells were fixed and incubated serially with a MAb (3H12 or 2H2), biotinylated anti-mouse IgG, ABC reagent, and VIP substrate.

Enzyme-linked immunosorbent assay (ELISA) for measuring IgG subclass antibody levels

Serum IgG subclass antibody levels to DENV-1 were determined by a conventional ELISA as described previously (Konishi et al., 2006). Briefly, 96-well microplates sensitized with rabbit hyperimmune serum against DENV-1 were incubated serially with: (i) DENV-1 antigen; (ii) two-fold serial dilutions (starting at 1:20) of each pooled serum sample; (iii) alkaline phosphatase-conjugated rabbit anti-mouse IgG1, IgG2a, IgG2b, or IgG3 (Rockland); and then (iv) p-nitrophenyl phosphate. The end-point titer was expressed as the maximum serum dilution that displayed an optic density (OD) value of $\geq$0.5, which was determined by the FORECAST function in Microsoft Excel (Microsoft Corporation). For cases in which the OD value was lower than 0.5 at the 1:20 serum dilution, the titer was defined as 1:10.

ELISA for measuring IgG concentrations

Concentrations of mouse IgG were determined by a sandwich ELISA as described previously (Yamanaka et al., 2008). Briefly, microplates sensitized with rabbit anti-mouse IgG polyclonal antibody (Bethyl) were incubated with two-fold serial dilutions of test samples, alkaline phosphatase-conjugated rabbit anti-mouse IgG1 or IgG2b (Rockland), and then p-nitrophenyl phosphate. Concentrations of mouse IgG1 or IgG2b were determined by comparing the OD with that obtained from reference mouse serum with a known concentration (Bethyl), using the FORECAST function in Microsoft Excel (Microsoft Corporation).

ELISA for measuring VLP expression

The VLP expression levels were determined by a sandwich ELISA. Microplates sensitized with rabbit anti-DENV-1 polyclonal antibody (Yamanaka et al., 2008) were sequentially incubated with test VLP samples, hyperimmune mouse ascitic fluids against DENV-1 (Konishi et al., 2006), alkaline phosphatase-conjugated rabbit anti-mouse IgG, and finally p-nitrophenyl phosphate. The relative ratios of VLP expressed by pcD1ME variants were determined by comparing the OD with that obtained for the original wildtype pcD1ME.

Preparation of single-round infectious particles (SRIPs)

SRIPs were prepared as described previously (Yamanaka et al., 2014). Briefly, 293T cells were co-transfected, using Lipofectamine LTX and Plus Reagent (Invitrogen), with two plasmids: (i) pCMV-JErep-fullC, which is a JEV replicon plasmid containing the JEV Nakayama strain (GenBank no. EF571853) full genome except for 1,971 nucleotides (positions 438–2,408)

corresponding to a portion of C not responsible for the synthesis of mature C, the full prM, and a major portion of E; and (ii) a pcDNA3-based plasmid expressing the prM and E (pcD1ME, pcBDV1 to pcBDV4, or their variants). Culture fluids were harvested after 3–6 days of incubation and used as SRIP antigens in the NAb/EAb-balance assay.

Statistical analysis

The statistical significance of differences was evaluated with a Student's *t*-test or the Mann–Whitney *U* test. Probability (p) less than 0.05 was considered significant.

Supplemental references

Konishi, E., and A. Fujii. 2002. Dengue type 2 virus subviral extracellular particles produced by a stably transfected mammalian cell line and their evaluation for a subunit vaccine. Vaccine. 20:1058-1067. doi:10.1016/S0264-410X(01)00446-7.

Konishi, E., M. Shoda, N. Ajiro, and T. Kondo. 2004. Development and evaluation of an enzyme-linked immunosorbent assay for quantifying antibodies to Japanese encephalitis virus nonstructural 1 protein to detect subclinical infections in vaccinated horses. J. Clin. Microbiol. 42:5087-5093. doi:10.1128/JCM.42.11.5087-5093.2004.

Konishi, E., K. Yagawa, and A. Yamanaka. 2008. Vero cells infected with vaccinia viruses expressing Japanese encephalitis virus envelope protein induce polykaryocyte formation under neutral conditions. Jpn. J. Infect. Dis. 61:410-411.

Konishi, E., Y. Tabuchi, and A. Yamanaka. 2010. A simple assay system for infection-enhancing and -neutralizing antibodies to dengue type 2 virus using layers of semi-adherent K562 cells. J. Virol. Methods. 163:360-367. doi:10.1016/j.jviromet.2009.10.026.

KEY RESOURCES TABLE

REAGENT or RESOURCE	SOURCE	IDENTIFIER
Antibodies		
Mouse monoclonal D1-V-3H12	Yamanaka et al. (2013)	N/A
Mouse monoclonal 3H12-IgG2b	Yamanaka et al. (2013)	N/A
Mouse monoclonal JE-2D5	Konishi et al. (2004)	N/A
Mouse monoclonal D1-4G2-4-15 (E specific, flavivirus group cross-reactive)	ATCC	HB-112
Mouse monoclonal D3-2H2-9-21 (prM specific, flavivirus group-cross-reactive)	ATCC	HB-114
Mouse monoclonal JE-10B4 (flavivirus-group-cross-reactive)	Konishi Laboratory	N/A
Rabbit hyperimmune polyclonal against DENV-1	This paper	N/A
Alkaline phosphatase-conjugated rabbit anti-mouse IgG1	Rockland	Cat#: 610-4540
Alkaline phosphatase-conjugated rabbit anti-mouse IgG2a	Rockland	Cat#:610-4541
Alkaline phosphatase-conjugated rabbit anti-mouse IgG2b	Rockland	Cat#:610-4542
Alkaline phosphatase-conjugated rabbit anti-mouse IgG3	Rockland	Cat#:610-4543
Rabbit anti-mouse IgG polyclonal antibody	Bethyl	Cat#: A90-217A
Biotinylated anti-mouse IgG	Vector Laboratories	Cat#: BA-2000
Bacterial and Virus Strains		
DENV-1 (Mochizuki strain)	Konishi Laboratory	N/A
DENV-2 (NGC strain)	Konishi Laboratory	N/A
DENV-3 (H87 strain)	Konishi Laboratory	N/A
DENV-4 (H241 strain)	Konishi Laboratory	N/A
Biological Samples		
Single-round infectious particles	Yamanaka et al. (2014)	N/A
Chemicals, Peptides, and Recombinant Proteins		
Mouse reference serum	Bethyl	Cat#: RS10-101
ABC (avidin-biotinylated peroxidase complex) reagent	Vector Laboratories	Cat#: PK6100
VIP substrate	Vector Laboratories	Cat#: SK4600
Low tox rabbit complement	Cedarlane	Cat#: CL3051
4-Nitrophenyl phosphate disodium salt hexahydrate	Sigma	Cat#: 71768

Critical Commercial Assays		
KOD -Plus- Mutagenesis Kit	TOYOBO	Cat#: SMK-101
Lipofectamine LTX and Plus Reagent	Invitrogen	Cat#: 15338-100
Experimental Models: Cell Lines		
Vero cells	Konishi Laboratory	N/A
K562 cells	Konishi Laboratory	N/A
Human embryonic kidney 293T cells	ATCC	Cat#: CRL-3216
Experimental Models: Organisms/Strains		
BALB/c mice	National Laboratory Animal Center, Mahidol University, Bangkok, Thailand	INB001
Oligonucleotides		
H2Nfw (5′-AATCTGACCACACGAGGGGGAGA-3′)	GeneDesign	N/A
H2Nrv (5′-GAACGCCAGGGCTGTGGGCA-3′)	GeneDesign	N/A
D87Nfw (5′-CAAAACGCGAACTTCGTGTGTCGACG-3′)	GeneDesign	N/A
D87Nrv (5′-TTCTTCCACCAGTGTGGCTTCTCCT-3′)	GeneDesign	N/A
L107Ffw (5′-GGGTTTTTCGGAAAAGGTAGCCTAAT-3′)	GeneDesign	N/A
L107Frv (5′-ACAGCCATTGCCCCAGCCTC-3′)	GeneDesign	N/A
Recombinant DNA		
Plasmid: pcDNA3	Konishi Laboratory	N/A
Plasmid: pcD1ME	Konishi et al. (2006)	N/A
Plasmid: pcD1-87	This paper	N/A
Plasmid: pcD1-107	This paper	N/A
Plasmid: pcD1-87/107	This paper	N/A
Plasmid: pcD1-2	This paper	N/A
Plasmid: pcD1-2/87	This paper	N/A
Plasmid: pcD1-2/107	This paper	N/A
Plasmid: pcD1-2/87/107	This paper	N/A
Plasmid: pcD1-D87F	This paper	N/A
Plasmid: pcD1-D87V	This paper	N/A
Plasmid: pcD1-D87E	This paper	N/A
Plasmid: pcD1-D87R	This paper	N/A
Plasmid: pcD1-D87Q	This paper	N/A
Plasmid: pcD1-L107P	This paper	N/A
Plasmid: pcD1-L107I	This paper	N/A
Plasmid: pcD1-L107W	This paper	N/A

Plasmid: pcD1-L107D	This paper	N/A
Plasmid: pcD1-L107K	This paper	N/A
Plasmid: pcD1-L107Q	This paper	N/A
Plasmid: pcBDV1	This paper	N/A
Plasmid: pcBDV1-87	This paper	N/A
Plasmid: pcBDV1-107	This paper	N/A
Plasmid: pcBDV2	This paper	N/A
Plasmid: pcBDV2-87	This paper	N/A
Plasmid: pcBDV2-107	This paper	N/A
Plasmid: pcBDV3	This paper	N/A
Plasmid: pcBDV3-87	This paper	N/A
Plasmid: pcBDV3-107	This paper	N/A
Plasmid: pcBDV4	This paper	N/A
Plasmid: pcBDV4-87	This paper	N/A
Plasmid: pcBDV4-107	This paper	N/A
pcBDVT (pcBDV1, pcBDV2, pcBDV3, pcBDV4)	This paper	N/A
pcBDVT-87 (pcBDV1-87, pcBDV2-87, pcBDV3-87, pcBDV4-87)	This paper	N/A
pcBDVT-107 (pcBDV1-107, pcBDV2-107, pcBDV3-107, pcBDV4-107)	This paper	N/A
pCMV-JErep-fullC	Yamanaka et al. (2014)	N/A
Software and Algorithms		
ICM-Browser	Molsoft, LLC	https://www.molsoft.com/download.html
ImmunoSpot 5.3.22 Professional DC software	Cellular Technology Limited	N/A
Other		
